# Prevalence of Chronic Conditions Among Medicare Part A Beneficiaries in 2008 and 2010: Are Medicare Beneficiaries Getting Sicker?

**DOI:** 10.5888/pcd11.130118

**Published:** 2014-01-16

**Authors:** Erkan Erdem

## Abstract

**Introduction:**

Medicare beneficiaries who have chronic conditions are responsible for a disproportionate share of Medicare fee-for-service expenditures. The objective of this study was to analyze the change in the health of Medicare beneficiaries enrolled in Part A (hospital insurance) between 2008 and 2010 by comparing the prevalence of 11 chronic conditions.

**Methods:**

We conducted descriptive analyses using the 2008 and 2010 Chronic Conditions Public Use Files, which are newly available from the Centers for Medicare and Medicaid Services and have administrative (claims) data on 100% of the Medicare fee-for-service population. We examined the data by age, sex, and dual eligibility (eligibility for both Medicare and Medicaid).

**Results:**

Medicare Part A beneficiaries had more chronic conditions on average in 2010 than in 2008. The percentage increase in the average number of chronic conditions was larger for dual-eligible beneficiaries (2.8%) than for nondual-eligible beneficiaries (1.2%). The prevalence of some chronic conditions, such as congestive heart failure, ischemic heart disease, and stroke/transient ischemic attack, decreased. The deterioration of average health was due to other chronic conditions: chronic kidney disease, depression, diabetes, osteoporosis, rheumatoid arthritis/osteoarthritis. Trends in Alzheimer’s disease, cancer, and chronic obstructive pulmonary disease showed differences by sex or dual eligibility or both.

**Conclusion:**

Analyzing the prevalence of 11 chronic conditions by using Medicare claims data provides a monitoring tool that can guide health care providers and policy makers in devising strategies to address chronic conditions and rising health care costs.

## MEDSCAPE CME

Medscape, LLC is pleased to provide online continuing medical education (CME) for this journal article, allowing clinicians the opportunity to earn CME credit.

This activity has been planned and implemented in accordance with the Essential Areas and policies of the Accreditation Council for Continuing Medical Education through the joint sponsorship of Medscape, LLC and Preventing Chronic Disease. Medscape, LLC is accredited by the ACCME to provide continuing medical education for physicians.

Medscape, LLC designates this Journal-based CME activity for a maximum of 1 **AMA PRA Category 1 Credit(s)™**. Physicians should claim only the credit commensurate with the extent of their participation in the activity.

All other clinicians completing this activity will be issued a certificate of participation. To participate in this journal CME activity: (1) review the learning objectives and author disclosures; (2) study the education content; (3) take the post-test with a 70% minimum passing score and complete the evaluation at www.medscape.org/journal/pcd (4) view/print certificate.

**Release date: January 16, 2014; Expiration date: January 16, 2014**

### Learning Objectives

Upon completion of this activity, participants will be able to:

- Evaluate previous research regarding trends in chronic medical conditions among adults
- Distinguish the general trend of the prevalence of chronic medical conditions in the current study
- Assess which chronic medical conditions are increasing in prevalence among older adults

**EDITORS**

Ellen Taratus, editor, *Preventing Chronic Disease*. Disclosure: Ellen Taratus has disclosed no relevant financial relationships.

**CME AUTHOR**

Charles P. Vega, MD, Associate Professor and Residency Director, Department of Family Medicine, University of California, Irvine. Disclosure: Charles P. Vega, MD, has disclosed no relevant financial relationships.

**AUTHORS AND CREDENTIALS**

Erkan Erdem, PhD, Senior Manager, KPMG, LLP, McLean, Virginia. Disclosure: Erkan Edem, PhD, has disclosed no relevant financial relationships.

## Introduction

Medicare beneficiaries who have chronic conditions are responsible for a disproportionate share of Medicare fee-for-service expenditures (1,2). The amount of research on rising health care costs and the deteriorating health of the US population is considerable. A recent study compared the percentage of people with 2 or more chronic conditions by using the National Health Interview Survey for 1999–2000 and 2009–2010. The study found that the percentage of people with 2 or more chronic conditions increased for adults aged 45 to 64, adults aged 65 or older, both men and women, all racial and ethnic groups, and most income groups between 1999–2000 and 2009–2010 (3). Having multiple chronic conditions is positively correlated with more physician visits, hospital admissions or days/nights spent in the hospital, and prescription medications (4). According to the Agency for Healthcare Research and Quality, health care for patients with multiple chronic conditions can cost up to 7 times as much as for patients with only 1 chronic condition (5). Recent increases in annual Medicare spending are associated mainly with the treatment of chronic conditions such as diabetes, arthritis, hypertension, and kidney disease (6). The objective of this study was to analyze the change in the health of the Medicare population between 2008 and 2010 by examining the prevalence of 11 chronic conditions in the Medicare fee-for-service population while controlling for age, sex, and dual eligibility.

## Methods

We conducted a retrospective analysis of the prevalence of chronic conditions among Medicare fee-for-service beneficiaries enrolled in Part A (hospital insurance). For the purposes of this study, the term “beneficiary” refers to anyone enrolled in Medicare, regardless of use of services (given that some beneficiaries never create a claim in a given year). The study used data from the 2008 and 2010 Chronic Conditions Public Use Files (PUFs); these 2 years are the only years for which these files are available from the Centers for Medicare and Medicaid Services (CMS) (7). The PUFs summarize administrative (claims) data for 100% of the Medicare fee-for-service population. They are aggregated files in which each record is a profile (or cell) defined by the characteristics of the Medicare beneficiaries: age, sex, various chronic conditions, and dual-eligibility status (ie, eligibility for both Medicare and Medicaid). Information is provided in 8 segments: Part A through Part D, each divided into 2 by length of enrollment (full year vs less than full year). In the segments for Part A (hospital insurance), Part B (medical insurance), and Part D (prescription drugs), many expenditure and utilization variables are provided as averages. For Part D, the available information is the total drug cost rather than Medicare payment. For people enrolled in Part C, or Medicare Advantage, the only available information in the PUFs is the number of people enrolled. Medicare Advantage plans are offered by private insurers who contract with Medicare to provide all Part A and Part B benefits; however, these insurers are not required to provide CMS with claims data. Beneficiaries of Part A, which is premium-free, are automatically enrolled in Part B. However, because Part B is voluntary and requires the payment of a monthly premium, beneficiaries can refuse enrollment. More than 90% of Part A beneficiaries are also enrolled in Part B, even though data on expenditures and utilization are summarized separately in the source files.

Data on the following 11 chronic conditions are available in the PUFs: Alzheimer’s disease and related disorders or senile dementia; cancer (breast, prostate, colorectal, and lung); congestive heart failure; chronic kidney disease; chronic obstructive pulmonary disease (COPD); depression; diabetes; ischemic heart disease; osteoporosis; rheumatoid arthritis/osteoarthritis; and stroke/transient ischemic attack. Because these PUFs are de-identified to be compliant with the privacy rules of the Health Insurance Portability and Accountability Act of 1996 (8), 6 of the chronic condition indicators (Alzheimer’s disease and related disorders or senile dementia, cancer, COPD, depression, osteoporosis, stroke/transient ischemic attack) are suppressed (or blanked) for about 0.4% of the Medicare fee-for-service population (8). Because the values for 6 chronic conditions are not provided for this group of beneficiaries, we could not determine whether the beneficiaries in such profiles had any of the 6 chronic conditions and excluded the profiles from the analyses.

The indicator for each chronic condition (whether or not a beneficiary has the condition) was determined by algorithms that search for a particular code in the beneficiary’s Medicare fee-for-service claims (9). These codes are the ICD-9 code (*International Classification of Diseases, Ninth Revision* [10]), the CPT-4 code (Current Procedural Terminology [11]), or the HCPCS code (Healthcare Common Procedure Coding System [12]). Hence, the PUFs are useful for investigating Medicare expenditures and utilization measures (eg, the number of hospital stays or visits) in Medicare programs for various types of care (eg, inpatient, outpatient) by chronic condition (1).

This study focused on only 1 segment in the PUFs: data on beneficiaries enrolled for a full year in Medicare Part A. We conducted descriptive analyses to study the health of this population. First, we calculated the average number of chronic conditions for each sex and each category of dual eligibility (dual-eligible and nondual-eligible) in 2008 and 2010 and calculated the percentage change. Second, we examined the change between 2008 and 2010 in the distribution of beneficiaries among categories defined by the number of chronic conditions (0, 1, and ≥2) for each sex, age group (<65 y, 65–69 y, 70–74 y, 75–79 y, 80–84 y, and ≥85 y) and category of dual eligibility. Third, we calculated the percentage point change between 2008 and 2010 in the prevalence of the 11 chronic conditions, by sex and category of dual eligibility. Because the analyses were based on 100% of the Medicare fee-for-service population (rather than a sample), the differences in percentages were not tested for significance. We performed the analyses using Stata version 12 (StataCorp LP, College Station, Texas).

## Results

The average number of chronic conditions increased by 2.0% between 2008 and 2010 (1.2% for nondual-eligible beneficiaries and 2.8% for dual-eligible beneficiaries) (Table 1). The increase in average number of chronic conditions for nondual-eligible women (1.5%) was larger than the increase for nondual-eligible men (1.0%). However, the opposite was true for dual-eligible beneficiaries: the increase in average number of chronic conditions for dual-eligible women (2.6%) was smaller than for dual-eligible men (3.8%). The increase in average number of chronic conditions for dual-eligible beneficiaries was larger than for nondual-eligible beneficiaries for both men (3.8% vs 1.0%) and women (2.6% vs 1.5%).

**Table 1:** Change in the Average Number of Chronic Conditions Among Medicare Part A Beneficiaries Between 2008 and 2010, by Sex and Dual Eligibility^a^

Eligibility	2008, n	2010, n	Change, %^b^
**All**			
All	1.435	1.464	2.0
Nondual	1.318	1.335	1.2
Dual	1.948	2.002	2.8
**Men**			
All	1.296	1.319	1.8
Nondual	1.243	1.256	1.0
Dual	1.574	1.633	3.8
**Women**			
All	1.549	1.583	2.2
Nondual	1.383	1.404	1.5
Dual	2.186	2.242	2.6

a Dual eligibility refers to eligibility for both Medicare and Medicaid. Source of data: Centers for Medicare and Medicaid Services (7).

b Percentages calculated from 2008 and 2010 values may not match because of rounding.

The prevalence of 2 or more chronic conditions increased between 2008 and 2010 for all sex–age categories except nondual-eligible men aged 65 to 69 (Table 2). For those aged 65 to 69, the prevalence of no chronic conditions increased by 1.6% for nondual-eligible men and 1.0% for nondual-eligible women, indicating an improvement in health among the newly enrolled. The increase in prevalence of 2 or more chronic conditions was the largest for beneficiaries younger than 65; this category consists mostly of people who are disabled or have end-stage renal disease. Among those younger than 65, the prevalence of no chronic conditions decreased among all categories of sex and dual eligibility: decreases ranged from 2.1% (nondual-eligible men) to 6.5% (all women). In this same age group, the prevalence of multiple chronic conditions increased among all categories of sex and dual eligibility: increases ranged from 4.6% (nondual-eligible men) to 7.0% (nondual-eligible women). 

**Table 2:** Percentage Change in the Distribution of Number of Chronic Conditions Among Medicare A Beneficiaries Between 2008 and 2010, by Sex, Age, and Dual Eligibility^a^

Age/No. of Chronic Conditions	Men, %			Women, %			All, %		
	All	Nondual	Dual	All	Nondual	Dual	All	Nondual	Dual
**<65 y**									
0	−3.6	−2.1	−4.4	−6.5	−4.9	−6.4	−4.8	−3.2	−5.4
1	1.9	1.1	1.3	1.2	2.0	−0.2	1.6	1.6	0.5
≥2	5.6	4.6	5.5	6.7	7.0	5.0	6.3	5.8	5.3
**65–69 y**									
0	1.5	1.6	0.2	0.7	1.0	−1.8	1.1	1.3	−0.7
1	−3.2	−3.3	−1.5	−2.2	−2.2	−2.0	−2.7	−2.8	−1.9
≥2	−0.1	−0.4	0.6	0.9	0.5	1.5	0.4	0.0	1.1
**70–74 y**									
0	0	0.3	−2.5	−0.3	0	−3.4	−0.1	0.2	−2.8
1	−2.1	−2.1	−1.3	−1.5	−1.3	−2.8	−1.8	−1.7	−2.3
≥2	1.5	1.4	1.7	1.4	1.1	1.9	1.5	1.2	1.8
**75–79 y**									
0	−0.3	0.1	−2.8	−1.5	−0.8	−4.3	−0.8	−0.3	−3.3
1	−1.6	−1.4	−1.6	−1.5	−1.0	−3.5	−1.5	−1.2	−2.9
≥2	1.1	0.8	1.4	1.7	1.2	1.9	1.4	1.0	1.7
**80–84 y**									
0	−1.3	−0.9	−2.1	−2.5	−1.9	−5.1	−1.9	−1.4	−3.4
1	−1.8	−1.6	−1.4	−1.9	−1.5	−3.2	−1.9	−1.6	−2.7
≥2	1.4	1.2	0.8	1.9	1.7	1.5	1.6	1.5	1.2
**≥85 y**									
0	−2.1	−1.9	−3.0	−3.5	−3.2	−5.1	−2.9	−2.7	−4.2
1	−2.8	−2.6	−3.4	−2.8	−2.4	−4.8	−2.8	−2.5	−4.6
≥2	1.8	1.8	1.0	2.1	2.3	1.3	2.0	2.1	1.2
**All**									
0	−0.3	0.4	−3.5	−1.1	−0.2	−3.9	−0.6	0.1	−3.4
1	−1.6	−2.0	0.3	−1.5	−1.5	−1.0	−1.6	−1.7	−0.4
≥2	1.4	0.9	2.7	1.7	1.2	1.7	1.5	1.1	1.9

a Dual eligibility refers to eligibility for both Medicare and Medicaid. Differences were not tested for significance because the analysis was based on claims for 100% of the Medicare fee-for-service population. Source of data: Centers for Medicare and Medicaid Services (7).

We found differences in the distribution of number of chronic condition between dual- and nondual-eligible beneficiaries for some age–sex categories (Table 2). Most of these differences indicated that dual-eligible beneficiaries became less healthy than nondual-eligible beneficiaries. For example, the prevalence of no chronic conditions decreased for dual-eligible men aged 70 to 74 and 75 to 79 and dual-eligible women aged 65 to 69 and 70 to 74 but increased for their nondual-eligible counterparts (except for women aged 70 to 74, for whom no change was found).

The changes were consistent (ie, they had the same mathematical sign, positive or negative) between men and women for most age categories (Table 2). When they differed, women generally became less healthy between 2008 and 2010 compared with men. For example, for beneficiaries aged 65 to 69, the prevalence of multiple chronic conditions among nondual-eligible men decreased, whereas it increased among nondual-eligible women. Similarly, for beneficiaries aged 75 to 79, the prevalence of no chronic conditions increased among nondual-eligible men, but it decreased among nondual-eligible women.

Overall, aggregation of data for sex and age categories (Table 2) indicated that the prevalence of multiple chronic conditions increased by about 1.5% between 2008 and 2010 (1.1% for nondual eligible beneficiaries and 1.9% for dual eligible beneficiaries). The prevalence of no chronic conditions decreased among all beneficiaries by 0.6% overall (increasing by 0.1% for nondual-eligible beneficiaries and decreasing by 3.4% for dual-eligible beneficiaries). Overall, the distribution of beneficiaries shifted from categories with no or 1 chronic condition to the category with multiple chronic conditions between 2008 and 2010.

The prevalence of the following conditions increased for both men and women between 2008 and 2010: chronic kidney disease, depression, diabetes, osteoporosis, and rheumatoid arthritis/osteoarthritis (Table 3). Also, the prevalence of the following conditions decreased for both men and women between 2008 and 2010: congestive heart failure, ischemic heart disease, and stroke/transient ischemic attack. The prevalence of cancer and COPD decreased for men but increased for women between 2008 and 2010. The changes in the prevalence of Alzheimer’s disease were mixed. It increased for all women and nondual-eligible men but decreased for dual-eligible men. Finally, the largest increase in prevalence among the 11 chronic conditions examined was for chronic kidney disease: 3.52 percentage points for all men (from 31.00% to 34.52%) and 3.88 percentage points for all women (from 25.22% to 29.10%).

**Table 3:** Percentage-Point Change in the Prevalence of 11 Chronic Conditions Among Medicare A Beneficiaries Between 2008 and 2010, by Sex and Dual Eligibility^a^

Chronic Condition	Men			Women			All		
	All	Nondual	Dual	All	Nondual	Dual	All	Nondual	Dual
Alzheimer’s disease and related disorders or senile dementia	−0.11	0.04	−0.30	0.07	0.02	0.13	0	0.03	−0.04
Cancer	−0.62	−0.72	−0.36	0.05	0.06	0.05	−0.27	−0.33	−0.12
Congestive heart failure	−1.84	−1.78	−1.93	−2.08	−2.00	−2.18	−1.98	−1.90	−2.08
Chronic kidney disease	3.52	3.54	3.50	3.88	3.88	3.87	3.72	3.72	3.72
Chronic obstructive pulmonary disease	−0.17	−0.18	−0.14	0.38	0.24	0.54	0.14	0.05	0.27
Depression	1.25	1.06	1.52	1.76	1.58	1.98	1.55	1.37	1.81
Diabetes	1.16	1.17	1.15	0.93	0.67	1.22	1.03	0.89	1.19
Ischemic heart disease	−0.57	−0.54	−0.63	−0.98	−1.02	−0.94	−0.82	−0.82	−0.82
Osteoporosis	0.28	0.21	0.44	0.14	0.02	0.32	0.18	0.07	0.34
Rheumatoid arthritis/osteoarthritis	0.88	0.55	1.41	1.11	0.89	1.36	1.02	0.75	1.37
Stroke/transient ischemic attack	−0.68	−0.55	−0.91	−0.70	−0.54	−0.90	−0.69	−0.55	−0.90

a Dual eligibility refers to eligibility for both Medicare and Medicaid. Differences were not tested for significance because the analysis was based on claims for 100% of the Medicare fee-for-service population (7).

## Discussion

This study compared the health of Medicare beneficiaries enrolled in Medicare Part A for a full year in 2008 and 2010 by using data on 11 chronic conditions from the CMS Chronic Conditions PUFs. Even though the average number of chronic conditions and the percentage of Medicare beneficiaries with multiple chronic conditions increased between 2008 and 2010, the prevalence of some of the chronic conditions (congestive heart failure, ischemic heart disease, and stroke/transient ischemic attack) decreased. Hence, the deterioration of health of the Medicare beneficiaries was predominantly due to other chronic conditions: chronic kidney disease, depression, diabetes, osteoporosis, and rheumatoid arthritis/osteoarthritis. Disaggregation of prevalence measures by sex showed differences in trends for other chronic conditions (ie, cancer and COPD).

Our study has several strengths. It highlights the chronic illnesses that affect a larger (or smaller) share of Medicare fee-for-service beneficiaries over time, regardless of sex and dual eligibility. Using data from Medicare fee-for-service beneficiaries, we not only show that this population is getting sicker on average (based on available information) but we also identify the chronic conditions that potentially increase the health care costs and decrease quality of life for millions of people.

Our study also has several limitations. First, the analysis compared the fee-for-service populations in only 2008 and 2010. The percentage of Medicare beneficiaries selecting Medicare Advantage plans increased from 22% to 24% between 2008 and 2010 (13). Migration to lower-cost plans may have left fewer healthy people in the Medicare fee-for-service population. Because CMS does not collect claims data from Medicare Advantage plans, we could not compare the health of those enrolled in Medicare Advantage with those in the Medicare fee-for-service.

Second, because the CMS Chronic Conditions PUFs included data on only 11 chronic conditions, we could not examine data on other diseases (eg, obesity and hypertension) that affect the health of Medicare beneficiaries. Our estimates (eg, average number of chronic conditions) might establish a lower limit for the actual average number of conditions because of conditions not included in the PUFs. However, because the 2008 and 2010 PUFs were created by using the same methods, our analyses provide reliable measures for the trend in the prevalence of the 11 chronic conditions.

Another limitation is that we used de-identified data. Six of the chronic condition indicators were suppressed for about 0.4% of the Medicare fee-for-service population because of privacy and confidentiality concerns (7), and we excluded these profiles from analyses. The suppression affected data on beneficiaries who had rare combinations of profile variables, including the very sick with multiple chronic conditions. All profiles for which chronic condition indicators were suppressed were profiles of beneficiaries who had at least 2 chronic conditions. Because a small number of profiles were excluded from our analysis, we would not expect the suppression of these data to influence the findings significantly.

Any strategy to control health care expenditures and improve the quality of life of older adults requires carefully designed policies and programs to prevent chronic illnesses and manage care for those who have them. This study demonstrates use of newly available Medicare PUFs that were developed by using claims data as an additional public health data source to identify and monitor prevalence trends in older adult Medicare fee-for-service beneficiaries. Use of these data for monitoring changes over time can help to inform health care providers and policy makers about trends in the prevalence of chronic diseases and investments in targeted initiatives and policies. Given the importance of monitoring trends, CMS recently began publishing chart books with more recent data (2011–2012) for a larger set of chronic condition indicators (including asthma, hypertension, and depression) than the set provided in the PUFs; this new information will allow researchers to continue analyzing the prevalence and burden of chronic conditions for the health care system over time (14,15).

## Figures and Tables

**Table:** 

**1. The activity supported the learning objectives.**				
**Strongly Disagree**				**Strongly Agree**
1	2	3	4	5
**2. The material was organized clearly for learning to occur.**				
**Strongly Disagree**				**Strongly Agree**
1	2	3	4	5
**3. The content learned from this activity will impact my practice.**				
**Strongly Disagree**				**Strongly Agree**
1	2	3	4	5
**4. The activity was presented objectively and free of commercial bias.**				
**Strongly Disagree**				**Strongly Agree**
1	2	3	4	5

## Post-Test Information

To obtain credit, you should first read the journal article. After reading the article, you should be able to answer the following, related, multiple-choice questions. To complete the questions (with a minimum 70% passing score) and earn continuing medical education (CME) credit, please go to http://www.medscape.org/journal/pcd. Credit cannot be obtained for tests completed on paper, although you may use the worksheet below to keep a record of your answers. You must be a registered user on Medscape.org. If you are not registered on Medscape.org, please click on the "Register" link on the right hand side of the website to register. Only one answer is correct for each question. Once you successfully answer all post-test questions you will be able to view and/or print your certificate. For questions regarding the content of this activity, contact the accredited provider, CME@medscape.net. For technical assistance, contact CME@webmd.net. American Medical Association's Physician's Recognition Award (AMA PRA) credits are accepted in the US as evidence of participation in CME activities. For further information on this award, please refer to http://www.ama-assn.org/ama/pub/about-ama/awards/ama-physicians-recognition-award.page. The AMA has determined that physicians not licensed in the US who participate in this CME activity are eligible for **AMA PRA Category 1 Credits™**. Through agreements that the AMA has made with agencies in some countries, AMA PRA credit may be acceptable as evidence of participation in CME activities. If you are not licensed in the US, please complete the questions online, print the AMA PRA CME credit certificate and present it to your national medical association for review.

## Post-Test Questions

### Article Title: Prevalence of Chronic Conditions Among Medicare Part A Beneficiaries in 2008 and 2010: Are Medicare Beneficiaries Getting Sicker?

**CME Questions**

1. You are seeing a married couple, both 70 years old. They bemoan the fact that they "have gotten sicker and sicker" and need to take increasing amounts of prescription drugs for multiple chronic medical conditions. According to previous data from the National Health Interview Survey, what can you tell them about the epidemiology of multiple chronic conditions among adults?
  - The percentage of people with 2 or more chronic medical conditions has increased only among adults 65 years and older
  - The percentage of people with 2 or more chronic medical conditions has increased among men, but not women
  - The percentage of people with 2 or more chronic medical conditions has increased only among minority racial and ethnic groups
  - There has been a broad increase in the percentage of people with 2 or more chronic medical conditions
2. Which of the following statements regarding trends in the number of chronic medical conditions in the current study of Medicare beneficiaries is ***most*** accurate?
  - There was no change in the number of chronic medical conditions between 2008 and 2010
  - The number of chronic medical conditions increased only among dual-eligible patients
  - The increase in the number of chronic medical conditions was more pronounced among dual-eligible vs nondual-eligible patients
  - There was a 14% increase in the number of chronic medical conditions among all individuals between 2008 and 2010
3. Which of the following medical conditions increased in prevalence between 2008 and 2010 in the current study?
  - Diabetes
  - Congestive heart failure
  - Alzheimer’s disease
  - Stroke/transient ischemic attack

**Evaluation**
